# Precise genetic engineering with *piggyBac* transposon in plants

**DOI:** 10.5511/plantbiotechnology.23.0525a

**Published:** 2023-12-25

**Authors:** Ayako Nishizawa-Yokoi, Seiichi Toki

**Affiliations:** 1 Institute of Agrobiological Sciences, National Agriculture and Food Research Organization (NARO), 3-1-3 Kannondai; 2 Graduate School of Nanobioscience, Yokohama City University, 22-2 Seto, Yokohama; 3 Faculty of Agriculture, Ryukoku University, 1-5 Yokotani, Seta Oe-cho, Otsu, Shiga 520-2194, Japan

**Keywords:** CRISPR/Cas9, DNA double-strand breaks, homologous recombination-mediated gene targeting, *piggyBac* transposon, rice

## Abstract

Transposons are mobile genetic elements that can move to a different position within a genome or between genomes. They have long been used as a tool for genetic engineering, including transgenesis, insertional mutagenesis, and marker excision, in a variety of organisms. The *piggyBac* transposon derived from the cabbage looper moth is one of the most promising transposon tools ever identified because *piggyBac* has the advantage that it can transpose without leaving a footprint at the excised site. Applying the *piggyBac* transposon to precise genome editing in plants, we have demonstrated efficient and precise *piggyBac* transposon excision from a transgene locus integrated into the rice genome. Furthermore, introduction of only desired point mutations into the target gene can be achieved by a combination of precise gene modification via homologous recombination-mediated gene targeting with subsequent marker excision from target loci using *piggyBac* transposition in rice. In addition, we have designed a *piggyBac*-mediated transgenesis system for the temporary expression of sequence-specific nucleases to eliminate the transgene from the host genome without leaving unnecessary sequences after the successful induction of targeted mutagenesis via sequence-specific nucleases for use in vegetatively propagated plants. In this review, we summarize our previous works and the future prospects of genetic engineering with *piggyBac* transposon.

## Introduction

Transposons were first discovered in maize as mobile genetic elements by Barbara McClintock (McClintock 1951) and have since been identified in the genomes of most organisms from bacteria to mammals. Taking advantage of the special properties of “jumping genes”, transposons have been widely employed for genetic engineering in processes such as transgenesis, insertional mutagenesis, and marker excision in a variety of organisms. In higher plants, the maize transposable element *Ac*/*Ds* (McClintock 1951) is the most widely used tool for functional genomics, even in heterologous plant species such as *Arabidopsis*, rice, tomato, carrot, potato, etc. (reviewed in Feschotte et al. 2002; Ramachandran and Sundaresan 2001). *Ac*/*Ds* are class II transposable elements, i.e. DNA transposons that move by a “cut-and-paste” mechanism. All DNA transposons ever found in various plant species leave a footprint at the excision site following every transposition event (reviewed in Döring and Starlinger 1986; Kidwell and Lisch 1997; Lönnig and Saedler 1997; Wessler 1988). Although they represent only minor nucleotide changes, such footprints left behind in protein-coding sequences or *cis*-elements required for gene expression can cause the introduction of frameshift mutations or alterations in gene expression.

**Figure 1:**
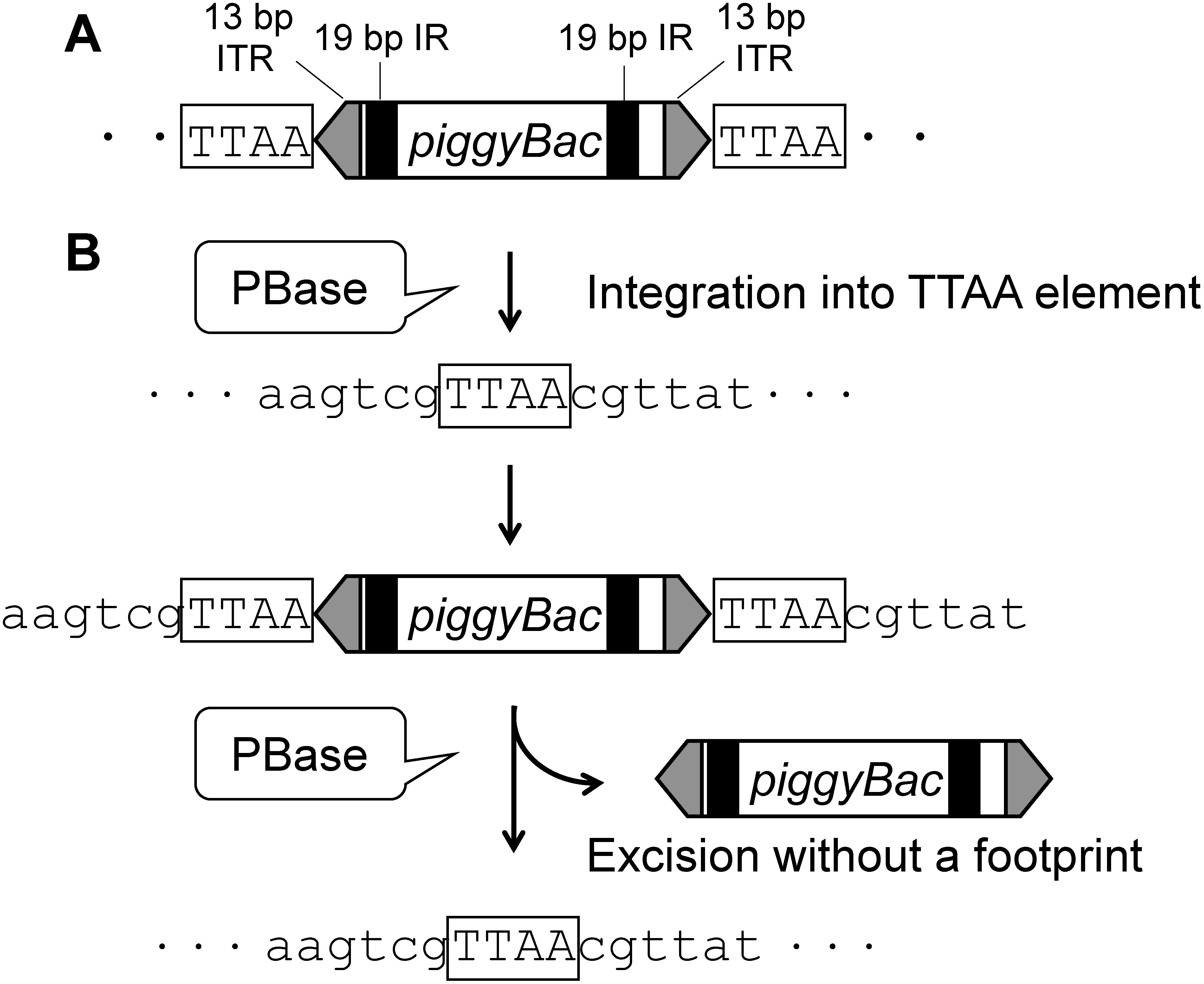
Figure 1. Schematic representation of the transposition reaction of *piggyBac* transposon. A, Diagram of *piggyBac* transposon. ITR, inverted terminal repeat; IR, sub-terminal inverted repeat. The *piggyBac* transposon is excised from the host genome by the expression of *piggyBac* transposase (PBase). B, Scheme of *piggyBac* transposition. PBase recognizes the terminal repeats and catalyzes transposition and integration into the TTAA element in the genome. The TTAA element is duplicated along the edges of inverted repeats of *piggyBac* during the integration of this transposon and returns to a single TTAA element in the subsequent excision step.

The *piggyBac* transposon derived from the lepidopteran cabbage looper moth *Trichoplusia ni* was originally isolated from baculovirus that was passaged in *T. ni* cells, that is, it was shown to jump from host *T. ni* cells into the baculovirus genome (Figure 1A) (Fraser et al. 1983). Unlike other DNA transposons, this transposon has the advantage that it can excise without leaving a footprint at the excised site (Cary et al. 1989; Fraser et al. 1996). The *piggyBac* transposon integrates into the host genome at a TTAA element; this TTAA element is then duplicated along the edges of inverted terminal repeats of *piggyBac* during integration of the transposon (Figure 1B) (Cary et al. 1989). Mitra et al. (2008) have demonstrated that the transposition of *piggyBac* does not require DNA synthesis, resulting in footprint-free transposition. Another important point is that the transposition of *piggyBac* is a simple mechanism thought to need only one protein, *piggyBac* transposase (PBase) (Cary et al. 1989). The *piggyBac* transposon comprises the PBase gene and inverted repeats containing 13-bp inverted terminal repeats and a 19-bp sub-terminal inverted repeat (Figure 1A). For genetic engineering with *piggyBac*, a two-element system is commonly used, that is, PBase and inverted repeats are split into a “helper” component and a “cargo” component carrying the gene of interest. Therefore, the *piggyBac* transposon has been used for transgenesis and insertional mutagenesis not only in a variety of insects (reviewed in Handler 2002) but also in many vertebrate cells, including human (Li et al. 2013), mouse (Wang et al. 2008), chicken (Liu et al. 2013), pig (Wu et al. 2013), etc. In our study, we have applied the *piggyBac* transposon to develop precise genome editing in plants.

## *piggyBac* transposes efficiently and accurately in plant cells

**Figure 2:**
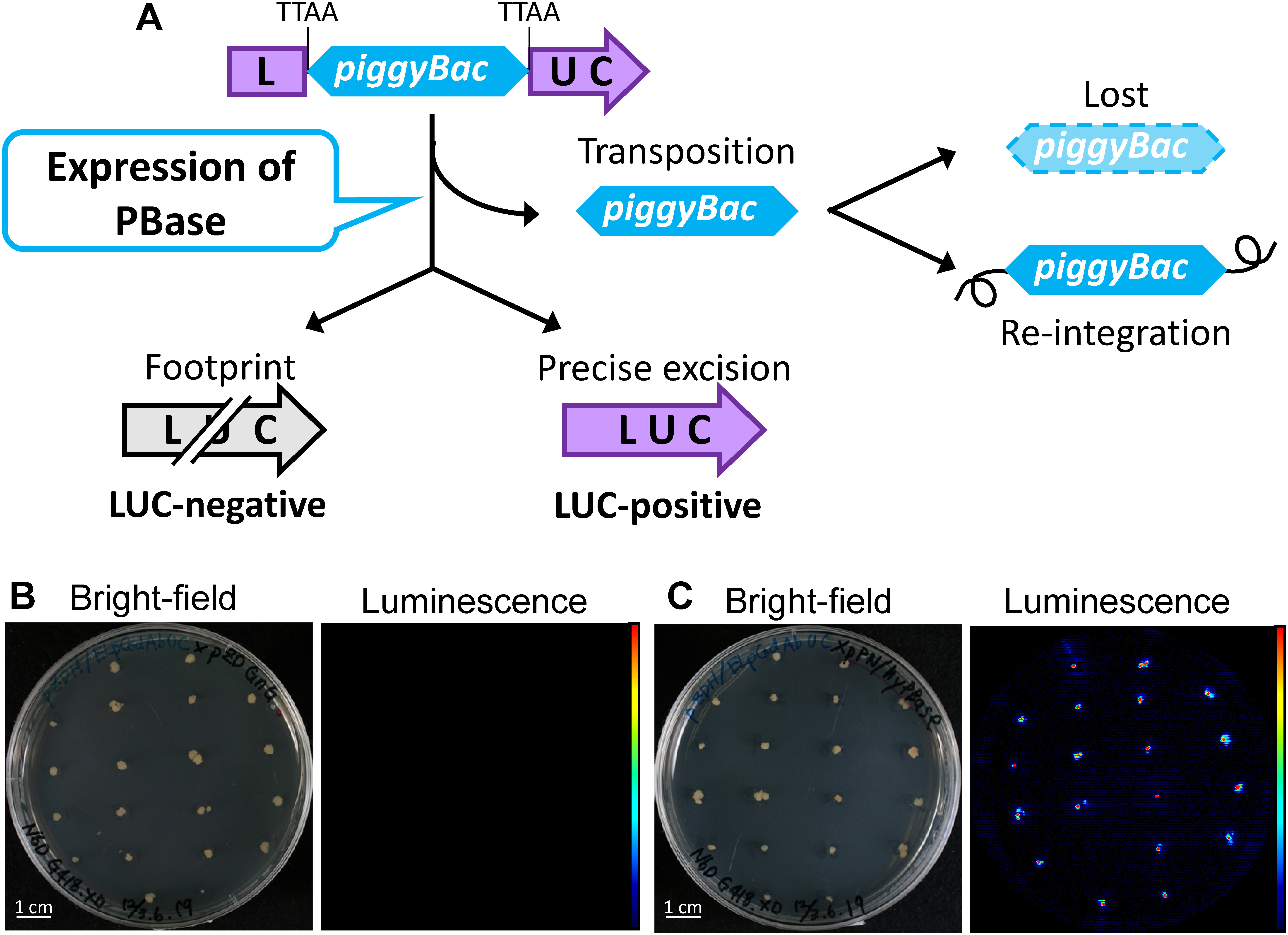
Figure 2. Efficient and precise transposition of *piggyBac* in PBase-expressing rice calli. A, Schematic representation of reporter constructs used to detect *piggyBac* transposition as luciferase luminescence in rice calli. Upon precise transposition of *piggyBac* from the luciferase (LUC) gene, transgenic calli become LUC-positive. B, C, Images of rice calli expressing GFP as a control (B) and hyPBase (C).

In the early 2010s, there was no evidence that a transposon derived from an animal, including insects, could transpose in plant cells. We developed a transposition assay system that allows *piggyBac* transposition to be visualized as luminescence derived from reconstituted luciferase expression cassettes in rice calli (Nishizawa-Yokoi et al. 2014). Reporter constructs carrying the luciferase (LUC) expression cassette containing the *piggyBac* transposon in the TTAA element of the LUC gene were introduced into rice calli via *Agrobacterium*-mediated transformation. If the *piggyBac* transposon is excised without leaving a footprint in the LUC gene, LUC luminescence can be detected in callus (Figure 2A). Transgenic calli harboring reporter constructs were then transfected with *piggyBac* transposase (PBase) expression cassettes in a second round of transformation. We used two types of PBase to evaluate the frequency of *piggyBac* transposition in rice calli. One is the insect *piggyBac* transposase (ePBase, Tamura et al. 2000) and the other is hyperactive *piggyBac* transposase (hyPBase). hyPBase, carrying 7 amino acid substitutions, was isolated and generated by screening a transposase mutant library in yeast and evaluating transposase activation in mouse ES cells (Yusa et al. 2011b). Yusa et al. (2011b) demonstrated that hyPBase is associated with increases of excision and integration of *piggyBac* of 17- and 9-fold, respectively, in mammalian cells.

After a 4-week selection period following a second round of transformation with transposase expression cassettes, LUC luminescence was detected in hyPBase and ePBase transgenic rice calli but not in control calli (Figure 2B, C). We observed that LUC luminescence in hyPBase-expressing rice calli was significantly higher than that in ePBase-expressing rice calli (Nishizawa-Yokoi et al. 2014). These findings indicated that the frequency of hyPBase-inducible transposition was higher than that of ePBase-inducible transposition in rice cells. In addition, we confirmed transposition of *piggyBac* from the LUC gene using PCR and Southern blot analysis, which revealed that the *piggyBac*-excised LUC fragment was detected either in hyPBase- or ePBase-expressing calli. Furthermore, sequence analysis of the *piggyBac*-excised LUC fragment showed that a TTAA element was restored after *piggyBac* transposition not only in hyPBase- but also in ePBase-expressing calli. The regenerated plants from LUC-positive calli expressing hyPBase were subjected to PCR analysis to analyze the frequency of *piggyBac* transposition from the reporter locus and of re-integration into another locus. More than 70% of regenerated plants lacked *piggyBac* from the reporter locus and half of them did not carry a re-integrated *piggyBac* transposon (Nishizawa-Yokoi et al. 2014). Recently, the *piggyBac* transposon was found to be active in tobacco (Johnson and Dowd 2014) and *Chrysanthemum morifolium* (Kishi-Kaboshi et al. 2023), although less efficiently compared with rice. These findings indicate that the *piggyBac* system can be widely applied to many plant species, and not only to monocotyledonous but also dicotyledonous plants.

## Precise marker excision using *piggyBac* transposon from the modified endogenous gene locus via homologous recombination-mediated gene targeting

Homologous recombination-mediated gene targeting (GT) is a precise genome engineering technique that makes possible the introduction of pinpoint modification into an endogenous target locus. In rice, the GT with positive-negative selection has been established as a reproducible method (Terada et al. 2002) and has been used to modify the various endogenous target genes (reviewed in Endo et al. 2018; Shimatani et al. 2014). Although positive-negative selection is a powerful tool for both the elimination of the transgenic cells carrying randomly integrated T-DNA and the enrichment of a small number of cells in which a GT event has occurred, the expression cassette of a positive selection marker gene inserted into the target locus together with the desired mutations should be removed completely to leave only the desired mutation in the target gene. Site-specific recombination systems such as Cre/*lox*P and transposons derived from plants such as *Ac*/*Ds* have been used widely to excise marker genes from the host genome in plants (reviewed in Hare and Chua 2002; Woo et al. 2011); however, using these methods, the residual sequences, e.g. the recognition sequences for the site-specific recombinase and a footprint for the transposon, are left at the marker excision site

**Figure 3:**
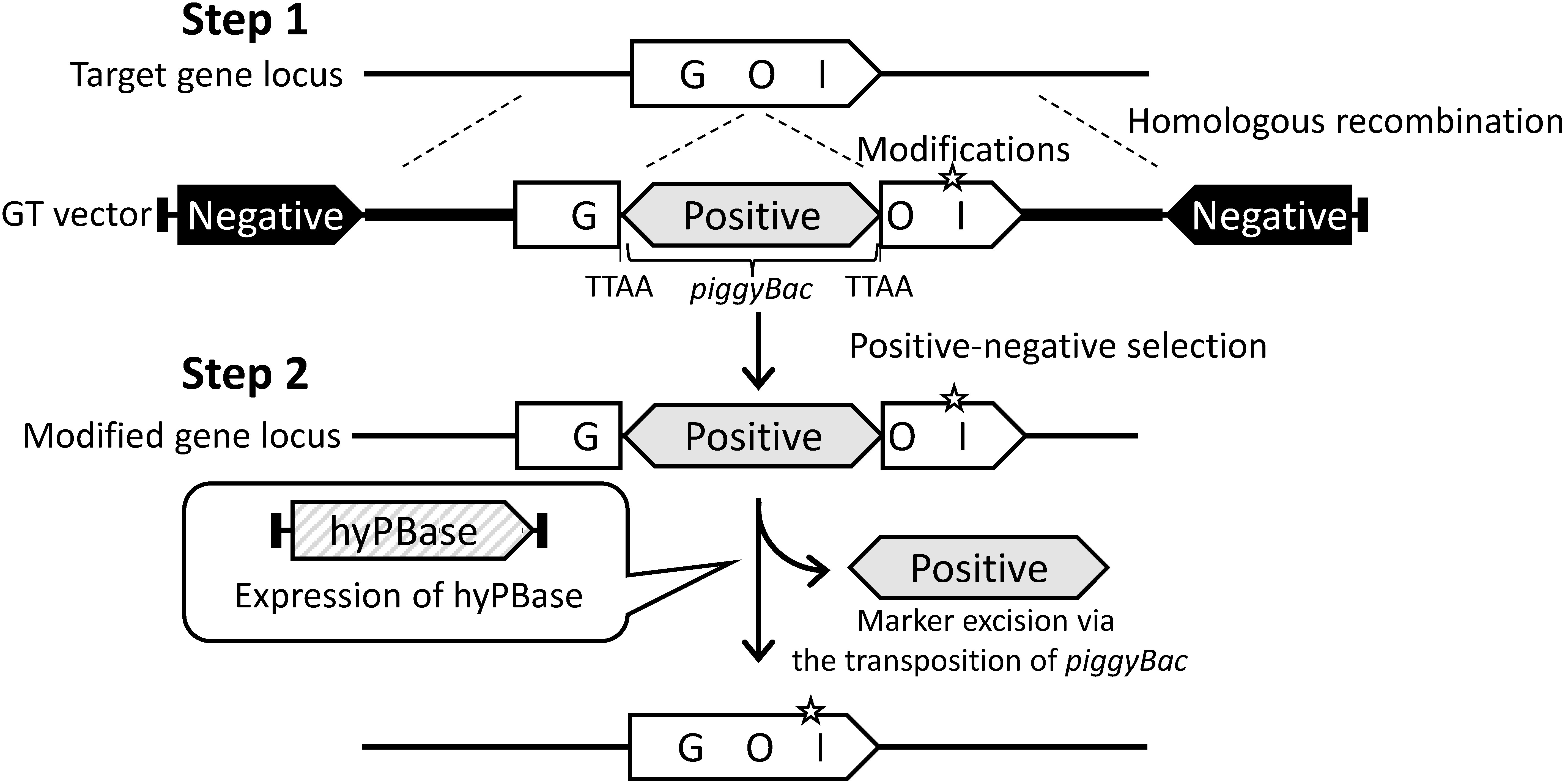
Figure 3. Strategy to introduce desired modifications into a gene of interest (GOI) via GT with positive-negative selection and subsequent marker excision from the GOI locus using *piggyBac* transposon. Step 1, Introduction of desired modifications (star) into the target gene via GT with positive-negative selection. The GT vector comprises the homologous sequence of target gene locus (white boxes and thick lines) with desired modifications (star), the *piggyBac* transposon carrying the positive selection marker (gray box), and the negative selection marker (black boxes). GT cells are enriched by positive-negative selection and are identified by PCR analysis. Step 2, Marker excision from the modified gene locus using *piggyBac* transposon. The expression cassette of hyPBase is transformed into GT calli to excise the positive selection marker via the transposition of *piggyBac*. If the *piggyBac* transposes without re-integration into another locus, the desired modifications are left behind in the target gene.

Yusa et al. (2011a) have reported that the *piggyBac* transposon could achieve precise marker excision from a target gene modified by GT in mammal cells without changing any nucleotide sequence. We also applied *piggyBac* transposon to excise a positive selectable marker gene from the target locus modified via GT with positive-negative selection in rice (Nishizawa-Yokoi et al. 2015a). At least in rice, a strong positive-negative selection system using the hygromycin phosphotransferase (*hpt*) gene as a positive selection marker and diphtheria toxin A subunit gene (*DT-A*) as a negative selection marker has been developed for the selection of the transgenic cells carrying the target gene modified via GT (Terada et al. 2002). As shown in Figure 3, the GT vector comprised the homologous sequence of the target locus with desired modifications, a negative selection marker cassette at both sides of the homologous sequence, and *piggyBac* transposon harboring the positive selection marker located within the homologous sequence. This GT vector was transformed into rice calli using *Agrobacterium*-mediated transformation. Transgenic calli were cultured on the selection medium and antibiotic-resistant calli were subjected to PCR analysis to identify GT-positive callus lines. The proportion of GT-positive calli per total antibiotic-resistant calli is approximately 1% because most antibiotic-resistant calli are false positives in which a truncated GT vector without the negative selection marker has integrated randomly into the genome. Following the isolation of GT-positive callus lines, an expression vector encoding hyPBase is introduced into GT-positive calli to remove the positive selection marker from target locus modified with GT via *piggyBac* transposition. The frequency of *piggyBac*-mediated marker excision is very high: more than 90% of regenerated plants from hyPBase expressing transgenic calli consistently display marker excision, with neither a footprint at the excised site nor re-integration into another locus.

The observed difference in *piggyBac* transposon re-integration frequency between transposition from the transgene locus of the LUC system described above and marker excision from an endogenous gene locus modified by GT is likely due to the difference in the culture period of rice calli used in transformation with the PBase expression vector. Before transformation of the PBase expression vector, transgenic calli transformed with the LUC system and GT calli were cultured on N6D medium for 8 and 14 weeks, respectively. It is well-documented that long-term tissue culture can lead to epigenetic changes genome-wide as well as the reactivation of transposons that had been epigenetically silenced (see reviews Smulders and de Klerk 2011; Springer and Schmitz 2017). Hence, re-integrated *piggyBac* transposon may excise again more easily in GT calli than in LUC transgenic calli. Under constitutive expression of PBase, *piggyBac* transposons are repeatedly re-integrated and excised in the genome, resulting in their complete loss from the genome. In addition, these transposons generally jump into gene loci close to the excised site. Therefore, the frequency of excision and re-integration will depend on the gene locus into which *piggyBac* originally integrated and its epigenetic state. Owing to the stable expression of hyPBase, re-integration of excised *piggyBac* transposon occurs in only a few percent of regenerated plants from hyPBase-expressing calli. Finally, homozygous plants carrying the modified target gene and segregated for having lost the hyPBase expression vector are obtained in the T_1_ generation.

GT with positive-negative selection and subsequent *piggyBac*-mediated marker excision has been applied successfully to the precise modification of any gene of interest at least in rice; several successful examples have been reported, such as the introduction of two amino acids substitutions into the *acetolactate synthase* (*OsALS*) gene, conferring herbicide tolerance (Nishizawa-Yokoi et al. 2015a); the introduction of a single base substitution into the microRNA target site of the *cleistogamy 1* (*OsCly1*) gene (Nishizawa-Yokoi et al. 2015a); and the changing of seven amino acids related to phosphorylation in the *SUPPRESSOR OF GAMMA RESPONSE1* (*OsSOG1*) gene (Nishizawa-Yokoi et al. 2023). Other than the introduction of pin-point mutations, this GT approach also makes it possible to introduce large modifications in the target gene, e.g. domain swapping, replacement of a coding region, exchange of *cis*-elements in a promoter region, and knock-in of reporter gene (Yoshioka et al. 2021).

## Establishing a *piggyBac*-mediated transgenesis system in rice

In addition to the advantage of excising without leaving a footprint at the excised site, *piggyBac* also has a very large cargo capacity, allowing 10–100 kb DNA fragments to be transposed in mammalian cells (Ding et al. 2005; Li et al. 2011). With these features, transgene-free induced pluripotent stem cells (iPSCs) have been established by the delivery of reprogramming factors and their subsequent excision after induction of reprogramming via *piggyBac* transposon in mammalian cells (Woltjen et al. 2009; Yusa et al. 2009). Similarly, we apply the *piggyBac* transposon to the delivery of transgenes in plant cells.

**Figure 4:**
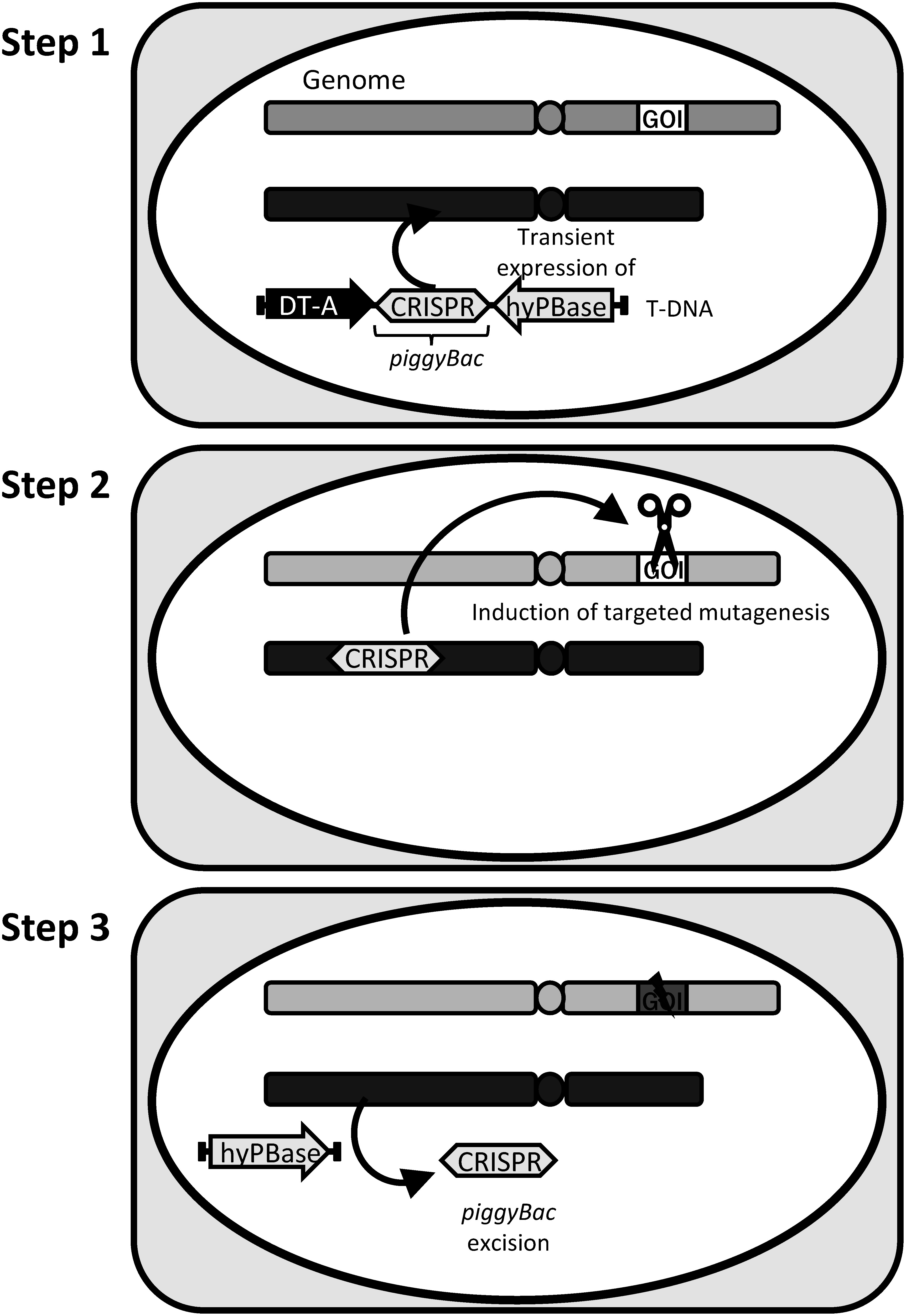
Figure 4. A strategy for *piggyBac*-mediated temporary transgenesis for the induction of targeted mutagenesis via CRISPR/Cas9 in plants. Step 1, The CRISPR/Cas9 expression cassette is integrated into the host genome from extrachromosomal T-DNA by *piggyBac* transposition, not by T-DNA integration, resulting from transient expression of hyPBase on T-DNA. Step 2, DNA double-strand breaks (DSB) and DSB-derived mutations are induced at the target gene by expression of CRISPR/Cas9. Step 3, *piggyBac* transposon is excised precisely from the host genome by expression of hyPBase, leaving only the targeted mutations in the target gene.

CRISPR/Cas9 (clustered regularly interspaced short palindromic repeats/CRISPR-associated protein 9, Jinek et al. 2012) is a tool used widely to generate mutant plants in basic research as well as in agricultural applications. It is especially desirable in agricultural applications to obtain mutant plants carrying only targeted mutations; therefore, DNA expression cassettes of CRISPR/Cas9 must be segregated out in the next generation. However, a transgene integrated into the genome is hard to eliminate via segregation in vegetatively propagated plants. We designed a *piggyBac*-mediated transgenesis approach that allows stable expression of a transgene integrated into the genome and subsequent complete excision from the host genome in plants (Figure 4) (Nishizawa-Yokoi and Toki 2021). As shown in Figure 4, CRISPR/Cas9 and selection marker expression cassettes within the *piggyBac* transposon are introduced into plant cells via *Agrobacterium*-mediated transformation, and these expression cassettes are integrated into the host genome from extra-chromosomal T-DNA via *piggyBac* transposition without T-DNA integration, following transient expression of hyPBase (Step 1). The single-strand T-DNAs imported into the plant cell nucleus are thought to replicate to a double-stranded form as extra-chromosomal T-DNAs, allowing transient expression of the transgene from T-DNA before integration into the host genome. Following expression of CRISPR/Cas9 from the transgene integrated into the genome, DNA double-strand breaks (DSBs) and DSB-derived mutations are induced at the target locus (Step 2). Finally, only the targeted mutations are left in the target gene through the transposition of *piggyBac* following transient expression of hyPBase (Step 3).

To establish the *piggyBac*-mediated transgenesis system in rice, we used a vector with a positive selection marker and CRISPR/Cas9 expression cassettes located within *piggyBac*, and transposase hyPBase and DT-A as a negative selection marker located outside of *piggyBac*. If the vector is integrated randomly into the genome, transgenic cells are excluded by the expression of DT-A. On the other hand, transgenic cells transformed via the transposition of *piggyBac* are concentrated by positive selection. Using this system, we demonstrated that the CRISPR/Cas9-expression cassette could be delivered from extrachromosomal T-DNA into the host genome in 1% of antibiotic-resistant rice calli via *piggyBac* transposition resulting from transient expression of hyPBase. DT-A, as well as PBase, was probably expressed transiently from extrachromosomal T-DNA in the transgenic cells, resulting in a decrease in cells transformed with CRISPR/Cas9-expression cassettes via the *piggyBac*-mediated transgenesis system. Therefore, we attempted to enhance the frequency of the *piggyBac*-mediated transgenesis system by improving the negative selection marker expression cassette, e.g., with the use of a conditional negative selection marker gene in which the negative selectable effect can be regulated by treatment of the transgenic cells with a specific agent. Subsequently, CRISPR/Cas9-mediated targeted mutations were detected at the target site in the *piggyBac*-mediated transgenic line. After temporary expression of CRISPR/Cas9, in a proof-of-concept experiment, we confirmed that the CRISPR/Cas9 expression cassette was excised completely from the genome via stably transformed hyPBase. To establish the *piggyBac*-mediated transgenesis system without leaving the transgene in the host genome, we are now attempting to excise the *piggyBac* transposon, once integrated into the genome, via transient expression of hyPBase. Efficient approaches for the transient expression of hyPBase include the use of agroinfiltration, a viral vector, and a chemical induction system.

## Prospects for further improvement of genetic engineering with *piggyBac* transposon

The application of precise gene modification via the combination of GT with positive-negative selection and *piggyBac*-mediated marker excision to a broad variety of plants remains challenging. To address this issue, we have used several approaches to establish a high-efficiency and universal GT method. The primary reason for the low efficiency of GT is thought to be that HR activity is very low in higher plants, resulting in the frequent occurrence of T-DNA random integration via non-homologous end joining (NHEJ) pathways competing for HR. Thus, we have demonstrated that suppression of the NHEJ-related factor, Ku70, Ku80, DNA ligase 4 (Lig4), and DNA polymerase theta led to a decreased frequency of T-DNA integration in rice (Nishizawa-Yokoi et al. 2012, 2021) and that rice *lig4* mutant calli exhibited enhanced HR and GT activity (Endo et al. 2016; Nishizawa-Yokoi et al. 2012). Moreover, our previous study showed that overexpression of the HR-related factors RecQl4 and/or exonuclease1 could enhance HR activity (Kwon et al. 2012) and that the treatment of rice calli with the HR-activated chemical (Rad51-stimulatory compound-1) could stimulate GT activity (Nishizawa-Yokoi et al. 2020). In addition to this approach, we attempted to establish a conditional negative selection marker gene to date there have been no successful reports of GT with positive-negative selection using the *DT-A* gene as a negative selection marker in other than rice. One of the conditional positive-negative selection systems that we established was a combination of *neomycin phosphotransferase II* (*nptII*) and an antisense *nptII* construct: the *nptII-anti nptII* system (Nishizawa-Yokoi et al. 2015b). A combination of our results and recent findings, e.g. efficient delivery of the GT template (Baltes et al. 2014; Butler et al. 2016; Cermak et al. 2015), and induction of DSBs at the target site via CRISPR/Cas9 (Endo et al. 2016; Nishizawa-Yokoi et al. 2020), etc., is expected to establish an efficient GT method widely applicable to various types of plant species.

Using *piggyBac* transposon, the frequency of marker excision from GT-modified gene was very high; however, transgenesis efficiency from extrachromosomal T-DNA is low even in rice. It has been reported that PBase mutants showing higher transposase activity compared with hyPBase were developed using a screening system in yeast and applied to mammalian cells (Wen et al. 2020). Furthermore, the fusion of PBase and TALE or CRISPR/Cas9 has been reported to enable the targeted integration and high-efficiency knock-in into a target gene in mammalian cells (Owens et al. 2013; Rezazade Bazaz et al. 2022). Recently, in mammalian cells, especially in hard-to-transfect cells like human iPSC, the *piggyBac*-mediated transgenesis system has made it possible to provide an effective gene correction approach using Prime Editor, which enables precise gene editing with the reverse transcription (Eggenschwiler et al. 2021; Wolff et al. 2021). We are trying to improve the *piggyBac*-mediated temporary transgenesis system using these approaches and hope to contribute to establishing efficient CRISPR/Cas9-mediated targeted mutagenesis and precise genome editing by prime editing in vegetatively propagated crops.

## Abbreviations

CRISPR/Cas9: clustered regularly interspaced short palindromic repeats/CRISPR-associated protein 9
DSBs: DNA double-strand breaks
*DT-A*: diphtheria toxin A subunit
ePBase: insect *piggyBac* transposase
GT: homologous recombination-mediated gene targeting
*hpt*: hygromycin phosphotransferase
hyPBase: hyperactive *piggyBac* transposase
LUC: luciferase
NHEJ: non-homologous end joining
*nptII*: *neomycin phosphotransferase II*
